# Paraxial Propagation of Scattered Light Based on the Chirp Z-Transform

**DOI:** 10.3390/s25051454

**Published:** 2025-02-27

**Authors:** Lujia Zhao, Yu-Ang Liu, Huiru Ji, Haibo Wang, Hao Tan, Yan Mo, Donglin Ma

**Affiliations:** 1 National Gravitation Laboratory, MOE Key Laboratory of Fundamental Physical Quantities Measurement, School of Physics, Huazhong University of Science and Technology, Wuhan 430074, China; zlj321000@163.com (L.Z.); hr.ji@foxmail.com (H.J.); whb2023@hust.edu.cn (H.W.); tanhao960410@hust.edu.cn (H.T.); moyan19950925@hust.edu.cn (Y.M.); 2 Institute of Optics, University of Rochester, 480 Intercampus Dr., Rochester, NY 14627, USA; 3 School of Optical and Electronic Information and Wuhan National Laboratory for Optoelectronics, Huazhong University of Science and Technology, Wuhan 430074, China; 4 Shenzhen Huazhong University of Science and Technology, Shenzhen 518057, China

**Keywords:** Wigner function, coherence, partially coherent light, chirp z-transform, scattering, light propagation

## Abstract

In the simulation of partially coherent light propagation within optical systems utilizing the Wigner function, the constraints imposed by the Fourier transform necessitate that the dimensions of the input and output matrices remain congruent. Consequently, the extent of the image plane is dictated by the dimensions of the light source matrix and the propagation distance. For optical systems of greater complexity, such simulations are highly memory-intensive. This paper innovatively incorporates the displacement theorem of the chirp z-transform and integrates it with the Wigner function. This approach affords enhanced flexibility in the simulation of partially coherent light transmission, enabling the targeted simulation of regions of interest within the frequency domain of the optical system, thereby significantly improving simulation efficiency. The efficacy of this novel method is demonstrated through the simulation of a Wigner transmission algorithm based on the chirp z-transform, applied to an RC (Ritchey–Chrétien) telescope system. The RC telescope, known for its optical design that minimizes aberrations and provides high-quality imaging, serves as a critical foundation for the simulation. The resultant simulations exhibit a high degree of consistency with traditional methods while offering increased flexibility, thus corroborating the validity and effectiveness of the proposed approach.

## 1. Introduction

Scattered light plays a pivotal role in numerous domains, including optical communication [1,2], medical imaging [3,4], and remote sensing detection [5,6]. The degradation of coherent light into partially coherent light due to scattering is a significant phenomenon, especially in optical systems with multiple scattering surfaces. As scattered light often results in the loss of coherence, it is crucial to simulate its propagation for various applications. The accurate simulation of scattered light transmission is thus of paramount importance in understanding light behavior under realistic optical conditions.

The simulation of scattered light involves studying the propagation of partially coherent light, which has been extensively explored in the literature. The propagation of partially coherent light through optical systems can be described using various mathematical methods, each offering distinct advantages for different scenarios. For instance, the Huygens–Fresnel diffraction integral represents a classical theory for beam propagation in free space [7]. Under the paraxial approximation, Collins integrated the Fresnel diffraction formula with the ABCD transformation matrix in matrix optics, thereby formulating the generalized Huygens–Fresnel diffraction formula [8] to delineate the propagation of light beams through optical elements. In non-paraxial scenarios, the beam transmission is characterized by the Rayleigh–Sommerfeld diffraction integral.

To address the challenges of simulating scattered light, several advanced mathematical methods have been proposed. These methods are essential for understanding and simulating the complex behavior of light as it propagates through scattering surfaces and optical systems.

In the context of optical scattering, methods such as least-squares wavelet analysis (LSWA) [9], S-transform, wavelet transform, and mode expansion have been used to enhance the precision and efficiency of scattering simulations.

The mode expansion method decomposes a partially coherent beam into mutually incoherent eigenmodes and calculates the propagation of each mode. In optical systems, after passing through multiple scattering surfaces, the coherence of the beam decreases, increasing the computational complexity [10]. This method helps to understand the propagation of light after it passes through scattering surfaces. LSWA [11] decomposes signals into different frequency components, helping to analyze surface roughness and scattering patterns of optical elements, thus predicting the behavior of scattered light more accurately. Wavelet transform enables multi-scale analysis [12], which is crucial for accurately characterizing light scattering on rough surfaces, particularly in analyzing the impact of mid-spatial frequency (MSF) and high-spatial frequency (HSF) roughness on light propagation. The S-transform [13] combines the advantages of Fourier and wavelet transforms, providing both time and frequency resolution [14,15]. It is used to capture the transient effects of scattered light, improving the accuracy of scattered light propagation simulations.

These advanced methods are especially relevant to the simulation of scattered light in optical systems, as they allow for a more detailed and accurate representation of surface roughness and scattering effects. While traditional methods like diffraction integrals and beam propagation models are widely used, they often become computationally complex when dealing with multiple scattering surfaces and high-frequency surface irregularities. The methods described above offer more efficient and flexible approaches for simulating scattered light, and they form the core of the simulation framework used in this paper.

In this study, the Wigner distribution is utilized to simulate the propagation of scattered light. The Wigner distribution, initially employed in the realm of mechanics, was subsequently introduced into the field of optics by Dolin and Walther [16]. The Wigner function is capable of characterizing the attributes of partially coherent light. The Wigner distribution can concurrently describe the distribution of light in both the spatial and angular domains [17], thus facilitating the coupling of the bidirectional scattering distribution function (BSDF) to depict light scattering, where BSDF is a characterization of the light scattering properties.

Traditional DFT-based Wigner simulations suffer from limitations in sampling flexibility. Once the number and spacing of sampling points are set on an object plane, the image surface size becomes fixed, and the number of sample points cannot be adjusted. This lack of flexibility significantly restricts the ability to simulate complex systems effectively, especially when varying sampling parameters are required. Furthermore, there are limited efficient methods available for simulating the propagation of partially coherent light in optical systems with multiple scattering surfaces.

To solve these problems, this study incorporates an RC (Ritchey–Chrétien) telescope system for the simulation. The RC telescope, widely used in astronomical and space optical systems, is known for its ability to reduce optical aberrations and provide high-quality imaging over a large field of view. Its unique design minimizes off-axis aberrations, which makes it an ideal choice for high-precision simulations of scattered light in complex optical systems. By using the RC telescope system in this study, we can validate the proposed method for more realistic and accurate light propagation simulations in optical systems with multiple scattering surfaces. The results demonstrate the method’s superior computational efficiency and flexibility compared to traditional approaches.

To address these challenges, the following contributions are made in this study:

1. The introduction of chirp z-transform (CZT) for simulating Wigner function propagation, offering flexible sampling capabilities and overcoming the limitations of traditional DFT-based methods.
2. By focusing on the specific frequency-domain regions of interest, the proposed method enhances simulation efficiency and significantly reduces memory usage by 50%.
3. The proposed method is validated through RC telescope simulations, showing a 43% improvement in runtime over DFT-based methods, demonstrating both computational efficiency and flexibility.

This paper is organized as follows: In Section 2, we introduce the Wigner function, chirp z-transform, and the theory of the Wigner function based on the CZT. In Section 3, we simulate the propagation of scattered light within optical systems. In Section 4, we compare and discuss the simulation results obtained using DFT and CZT. Section 5 concludes the paper with a summary and outlook.

## 2. Theory

In this chapter, we delve into the propagation theory of partially coherent light, which is based on the Wigner function and the application of the chirp z-transform.

### 2.1. The Wigner Function

For partially coherent light, the Wigner distribution is obtained through the Fourier transform of the cross-spectral density function of the light. The cross-spectral density function of a partially coherent light field $E\left(x\right)$ can be mathematically represented as:

(1) $$\Gamma (x,\Delta x)=\langle E\left(x+\frac{\Delta x}{2}\right)E^{*}\left(x-\frac{\Delta x}{2}\right)\rangle$$

where 〈 〉 denotes ensemble average, Δx is the distance between two points and the asterisk is complex conjugation. The Wigner function then can be defined as [18]:

(2) $$W(x,u)=\int \Gamma (x,\Delta x)\exp\left(-i\frac{2\pi }{\lambda }u\Delta x\right)d\Delta x$$

where *u* signifies the spatial frequency, and *x* denotes the spatial coordinate. It is evident from the equation that the Wigner function is capable of concurrently characterizing the distribution of light in both the spatial and angular domains. This dual-domain representation facilitates the integration of light scattering models. In accordance with the intrinsic properties of the Wigner function, the correlation between the Wigner function and the light intensity of the field I(x) is articulated as follows:

(3) $$I(x)=\frac{1}{2\pi }\int W(x,u)du$$

### 2.2. Propagation of Wigner Function in Free Space

In the paraxial approximation, according to the linear optical transmission theory, the input and output relationship of the optical system is [19]:

(4) $$W_{o}(x_{o},u_{o})=\gamma W_{i}(Ax_{i}+Bu_{i},Cx_{i}+Du_{i})$$

where the subscripts *o* and *i* represent input and output, respectively, and constant γ is non-negative, which equals the unit 1 when an optical system is lossless. We can represent the above equation with a transfer matrix:

(5) $$\left[\begin{array}{c} x_{o}\\ u_{o} \end{array}\right]=\left[\begin{array}{cc} A & B\\ C & D \end{array}\right]\left[\begin{array}{c} x_{i}\\ u_{i} \end{array}\right]$$

According to the geometric optics principle, the transfer matrix in free space becomes:

(6) $$\left[\begin{array}{c} x_{o}\\ u_{o} \end{array}\right]=\left[\begin{array}{cc} 1 & z\\ 0 & 1 \end{array}\right]\left[\begin{array}{c} x_{i}\\ u_{i} \end{array}\right]$$

where *z* is the propagation distance. Thus, the Wigner distribution in free space under paraxial optics can be obtained as:

(7) $$W_{o}\left(x_{o},u_{o}\right)=W_{i}\left(x_{i}+u_{i}z,u_{i}\right)$$

As can be seen from the above formula, the transmission of Wigner distribution in free space is a shearing process in the *x* direction. Using the displacement theorem of Fourier transform [20], Equation (7) can be expressed in terms of Fourier optical properties [21]:

(8) $$W_{o}\left(x,u\right)=FT^{-1}\left\{\exp(-i2\pi uzv)FT\left\{W_{i}\left(x,u\right)\right\}\right\}$$

where v is the Fourier transform pair of x. According to Equation (8), the propagation of the Wigner function in free space is actually a Fourier transform process.

### 2.3. Wigner Function in Optical Systems

Suppose the amplitude and phase modulation of the optical element is t(x), the complex amplitude of the light field through the thin element can be calculated as [22]:

(9) $$E_{o}(x)=t(x)E_{i}(x)$$

Substitute the above equation into Equations (1) and (2), the Wigner distribution of light field after passing through the optical element can be obtained as:

(10) $$W_{o}\left(x,u\right)=\int \langle E\left(x_{i}+\frac{\Delta x}{2}\right)E^{*}\left(x_{i}-\frac{\Delta x}{2}\right)t\left(x+\frac{\Delta x}{2}\right)t^{*}\left(x_{i}-\frac{\Delta x}{2}\right)\rangle \exp\left(-i\frac{2\pi }{\lambda }u\Delta x\right)d\Delta x$$

Simplifying the above equation yields Equation (11), as shown below:

(11) $$W_{o}\left(x,u\right)=\int \Gamma (x_{i},\Delta x)\Gamma_{t}(x_{i},\Delta x)\exp\left(-i\frac{2\pi }{\lambda }u\Delta x\right)d\Delta x$$

where $\Gamma_{t}$ is the cross spectral density function of thin element, and [23]

(12) $$t(x)=\exp\left[j2\pi \left(n_{2}\cos u_{o}-n_{1}\cos u_{i}\right)h(x)\right]$$

where $u_{i}$ and $u_{o}$ are the incidence angle and the scattering angle, respectively. *n*_1_ and *n*_2_ are the refractive index after scattering. h(x) is the profile height distribution of the optical surface.

By substituting the definition of Wigner function and applying the convolution theorem of Fourier transform, the Wigner distribution of the scattered field can be written as [22]:

(13) $$W_{o}(x,u)=W_{i}(x,u)⊗W_{t}(x,u)$$

where $W_{i}(x,u)$ and $W_{t}(x,u)$ are Wigner distributions of incident field and scattered field, respectively, and ⊗ represents the convolution of the angular domain. Therefore, the Wigner distribution of the light field after the scattered surface can be calculated as the Wigner distribution of the incident light convolved with the Wigner distribution of the surface transmittance function.

Assuming that the surface profile is a generalized stationary Gaussian process, combined with paraxial approximation, the Wigner distribution of the scattering surface can be obtained as [24]:

(14) $$W_{t}(x,u)=(1-\mathrm{TIS})\delta (u)+S(u)$$

where TIS is the total integrated scatter and S(u) is the angle distribution function, which is positively correlated with the power spectral density (*PSD*) of the surface profile:

(15) $$S(u)=\frac{4\pi^{2}{\left(n-1\right)}^{2}}{\lambda^{4}}PSD\left(\frac{u}{\lambda }\right)$$

Therefore, the Wigner distribution of the light field after the scattering surface is:

(16) $$W_{o}(x,u)=(1-TIS)\cdot W_{i}(x,u)+W_{i}(x,u)⊗S(u)$$

For the general case, the Wigner function of scattered light should be calculated by integrating *BSDF* against the incidence angle:

(17) $$W_{o}(x,u)=(1-TIS)\cdot W_{i}(x,u)+\int_{-\pi /2}^{\pi /2}W_{i}\left(x,u\right)BSDF\left(u_{i},u\right)du_{i}$$

where BSDF is the result of normalization of the irradiance of the incident surface and the radiance of the scattered surface. For the case where surface roughness *RMS* is much smaller than wave length, the relationship between *PSD* and *BSDF* is determined by the Rayleigh–Rice perturbation theory [25]:

(18) $$BSDF\left(u_{i},u\right)=\frac{4\pi^{2}\Delta n^{2}}{\lambda^{4}}\cos u_{i}\cos uPSD\left(f\right)$$

where Δn is the difference in refractive index between the two sides of the scattering surface, f stands for the spatial frequency of the optical surface, and *PSD* is the function of f. For most optical surfaces whose surface roughness is much smaller than the wavelength, the *PSD* can be approximated by the K-correlation model [25]:

(19) $$PSD(f)=A{\left[1+{\left(Bf\right)}^{2}\right]}^{-C/2}$$

where *A* is the size of the *PSD* at low frequency, 1/*B* is the spatial frequency when the “roll down” occurs, proportional to the surface space wavelength (also known as the autocorrelation length), *C* is the slope of the *PSD* when the spatial frequency is greater than 1/B.

The *BSDF* of the optical surface can be obtained by substituting Formula (19) into Formula (18).

The Wigner distribution of partially coherent light in an optical system can be computed using Equations (9)–(19). For optical components, we calculate their transmission function and use Equations (10) and (11) to determine the Wigner distribution. In practical applications, the surfaces of optical elements are inherently imperfect. Based on established theories, surface imperfections can be categorized into figure errors, mid-spatial frequency (MSF) errors, and high-spatial frequency (HSF) errors, also referred to as micro-roughness [26]. Micro-roughness errors typically manifest as wavefront aberrations and, therefore, are not considered in this paper. HSF causes wide-angle scattering, while MSF results in small-angle scattering. Since MSF errors are generally introduced during manufacturing, their transmission function can be modeled, allowing the Wigner distribution to be calculated using Equations (10) and (11). In contrast, HSF is usually characterized by a surface *PSD*, and its Wigner distribution is computed using Equation (17).

As discussed above, the calculation of the Wigner distribution requires the Fourier transform. According to the characteristics of Fourier transform, the number and interval of sampling points in the spatial and frequency domains must be equal, which greatly limits the application of the algorithm. Thus, in order to save the computer memory, the number and interval of sampling points are selected flexibly, and the chirp z-transform is used to replace the Fourier transform.

### 2.4. The Chirp Z-Transform

When dealing with sampled data, the *z*-transform is equivalent to the Laplace transform in a continuous time system. The *z*-transform of the sequence $x_{n}$ on *N* finite points can be expressed as [27]:

(20) $$X(z)=\sum_{n=0}^{N-1}x_{n}z^{-n}$$

Take a finite *z* value, which is $z_{k}$, and thus the above equation becomes:

(21) $$X_{k}=X\left(z_{k}\right)=\sum_{n=0}^{N-1}x_{n}z_{k}^{-n}$$

The chirp z-transform is defined by the above equation. When *z_k_* = exp(*j*2π*k*/*N*), *k* = 0,1,…, *N* − 1, the chirp z-transform becomes DFT:

(22) $$X_{k}=\sum_{n=0}^{N-1}x_{n}\exp(-j2\pi k/N),k=0,1,\cdots ,N-1$$

In Equation (22), the contour of the sampling point on the *z*-plane is delineated as a unit circle with equidistant spacing. However, in a more general context, the profile of the sampling points in the *z*-plane assumes a helical configuration, with the sampling points exhibiting uneven spacing. Here, we have [28]:

(23) $$z_{k}=AW^{-k},k=0,1,\cdots ,M-1$$

where

(24) $$A=A_{0}e^{j2\pi \theta_{0}}$$

(25) $$W=W_{0}e^{j2\pi \phi_{0}}$$

where the parameter *A* governs the initial sampling point in the chirp z-transform, *W* dictates the sampling interval, and *K* determines the total number of sampling points. It should be noted that the parameter *M* does not necessarily have to be an integer power of 2, but for computational convenience, an integer power of 2 is often selected. By adjusting the parameters *M*, *A*, and *W*, the chirp z-transform algorithm can efficiently compute the z-transform of a sequence with arbitrary starting and ending positions, as well as any sampling interval in the z-plane, and obtain an output sequence of length *M*. Consequently, the chirp z-transform offers a higher degree of flexibility compared to the Fourier transform.

### 2.5. Application of the Chirp Z-Transform in Wigner Propagation

As shown above, the transmission of the Wigner distribution of light in free space is a shearing process. To achieve this simulation, we use the Fourier shift theorem, and therefore we apply the shift theorem to the chirp z-transform, i.e.,

(26) $$\mathrm{CZT}[x(n-n_{0})]=\mathrm{CZT}[x(n)]z^{n_{0}}=\mathrm{CZT}[x(n)]A^{-n_{0}}W^{kn_{0}}$$

Hence, for a Wigner distribution with a propagation distance of *z*, the chirp z-transform is implemented as:

(27) $$W_{o}\left(x,u\right)={\mathrm{ICZT}}^{-1}\left\{A^{-n_{0}}W^{kn_{0}}\mathrm{CZT}\left\{W_{i}\left(x_{i},u\right)\right\}\right\}$$

Utilizing Equation (27), we possess the capability to freely manipulate the sampling process during beam propagation. Analogous to the inverse discrete Fourier transform (DFT), the inverse chirp z-transform (ICZT) also encounters high-frequency oscillations attributable to frequency misalignment. To address this issue, we modify the sampling direction of the signal by inverting the parameter *A* and taking the complex conjugate of the parameter *W*. Specifically, this entails the following adjustments:

(28) $$A^{\prime }=-A_{0}e^{j2\pi \theta_{0}+\pi }=-A$$

(29) $$W^{\prime }=W_{0}e^{-j2\pi \phi_{0}}=W^{*}$$

By substituting the above parameters into Equation (27), we can replace the DFT with CZT.

## 3. Simulation of Partially Coherent Light Transmission

In this chapter, we delve into a classical telescope system, specifically the Ritchey–Chrétien (RC) telescope system, for the purpose of simulation. By meticulously controlling the surface roughness of the telescope, we are able to accurately determine the transmission characteristics of scattered light within the optical system.

### 3.1. Optical System and Simulation Parameters

**Figure 1:**
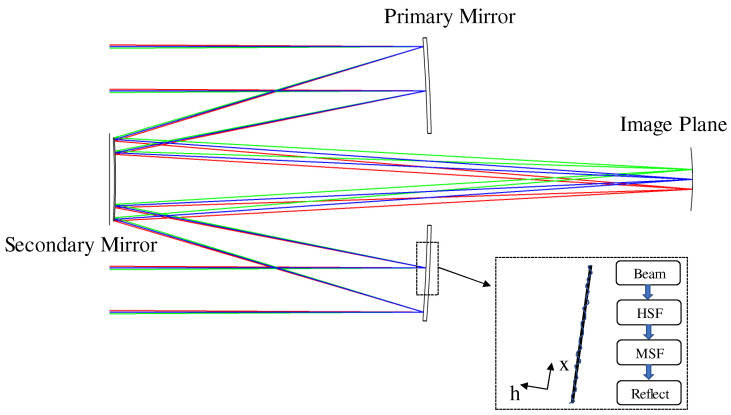
The profile of the RC telescope system.

Figure 1 shows the overall structure of the RC telescope system, with an entrance pupil diameter of 18.75 mm. Partially coherent light, originating from an effectively infinite distance, strikes the primary mirror. Due to the HSF and MSF errors on the surface of the primary mirror, the incident light successively modulated by HSF and MSF before being reflected. After reflection, the light propagates through free space, during which it is further influenced by the surface roughness of the secondary mirror. Ultimately, the light traverses free space once more before reaching the image plane.

**Figure 2:**
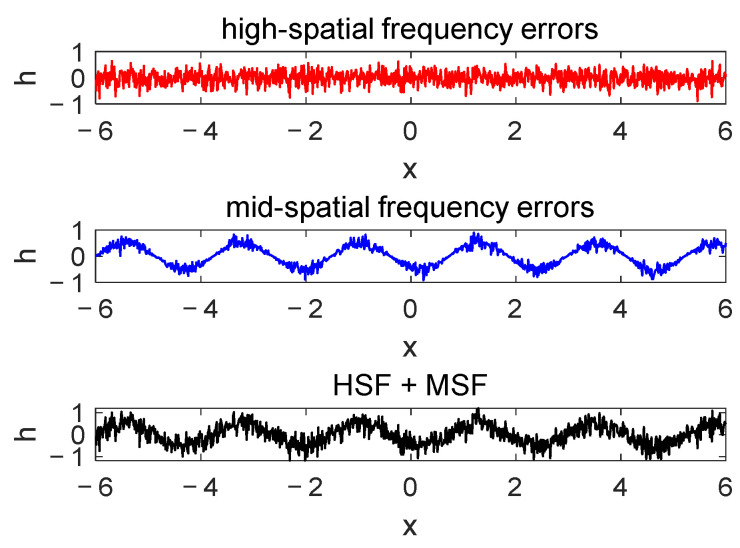
The profile of the optical surface error.

As shown in Figure 1, a portion of the primary mirror is highlighted by a dotted frame, with a magnified view of this area provided on the right. The figure illustrates the surface roughness error (represented by the red line) on the primary mirror. The horizontal coordinate h represents the contour height of the optical surface, while the vertical coordinate x indicates the position along the surface. A more detailed roughness profile of the primary mirror is provided in Figure 2. The HSF error represents unpredictable high-frequency errors, while the MSF error is a periodic error arising from the manufacturing process.

In this paper, we use a multi-Gaussian Schell model beam with a diameter of 5 mm and a wavelength of 0.633 μm for the simulation [29]. The beam has an amplitude distribution similar to that of a flat-topped Gaussian beam, and the coherence is Gaussian. By calculating the cross-spectral density function of the multi-Gaussian Schell model beam, as shown in Equations (1) and (2), the Wigner distribution of the light source can be obtained.

The simulation parameters are shown in Table 1.

### 3.2. Simulation Result

**Figure 3:**
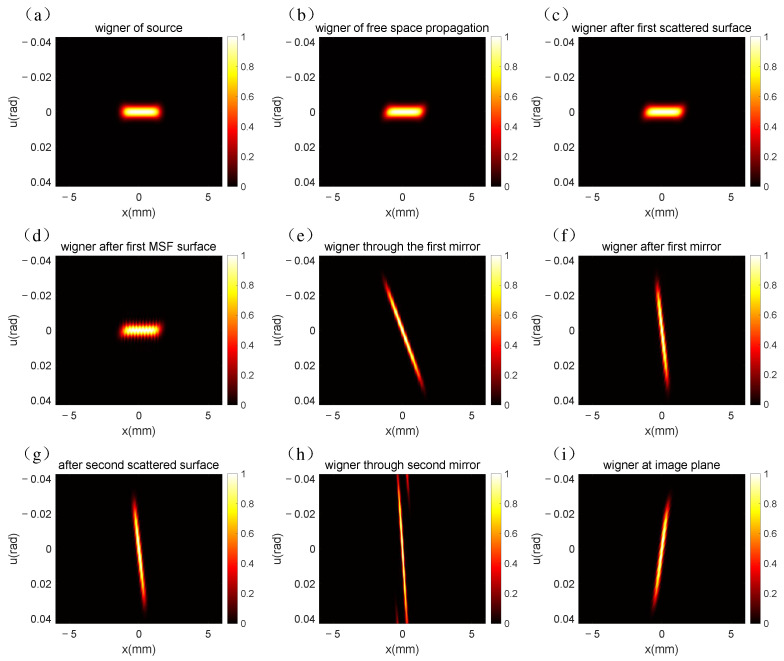
The DFT-based Wigner distribution in the RC optical system: (**a**) Wigner distribution of the multi-Gauss Schell model beam; (**b**) Wigner distribution after free space propagation; (**c**) Wigner distribution HSF errors over the surface of the primary mirror; (**d**) Wigner distribution MSF errors over the surface of the primary mirror; (**e**) Wigner distribution of phase modulated by primary mirror; (**f**) free space transmission from primary mirror to secondary mirror; (**g**) Wigner distribution HSF errors over the surface of the secondary mirror; (**h**) Wigner distribution of phase modulated by primary mirror; (**i**) free space transmission from secondary mirror to the image plane.

The results of the propagation simulation from the source to the image plane using the Wigner function are shown in Figure 3.

As can be seen from Figure 3a, the Wigner distribution of the multi-Gaussian Schell model beam is uniform. By using Equation (8), we can obtain the Wigner distribution of the partially coherent light source after propagation through free space, as shown in Figure 3b. Compared to Figure 3a, the shape of Figure 3b is closer to a parallel quadrilateral, which is because the Wigner distribution of the light source is shear transformed when it is transmitted in free space. According to Equation (17), Figure 3c,g show the influence of HSF errors of primary and secondary mirrors on the Wigner distribution of light, respectively. It is clear that the Wigner distribution becomes wider in Figure 3c because the scattered light caused by HSF makes the spot larger. However, the change in Figure 3g is not so significant because we have a coherence length of 100 μm on the surface of the primary mirror and a coherence length of 250 μm on the surface of the secondary mirror, so the HSF scattering of the secondary mirror is smaller than that of the primary mirror. Figure 3d denotes the MSF error of the primary mirror, which is obtained using Equations (10) and (11). The Wigner distribution oscillates violently, which is caused by the intermediate frequency error of the surface. Here, we have introduced a periodic error, so Figure 3d is periodic. Both Figure 3e,h represent the phase modulation of the aspherical mirror. It is calculated from the optical path difference generated by the mirror. The change in Wigner distribution direction is due to the fact that the phase modulation of the mirror changes the wave vector of the light. It can be seen from Figure 3i that the Wigner distribution has a significant shear, which is due to the long distance of light transmission from the secondary mirror to the image plane.

**Figure 4:**
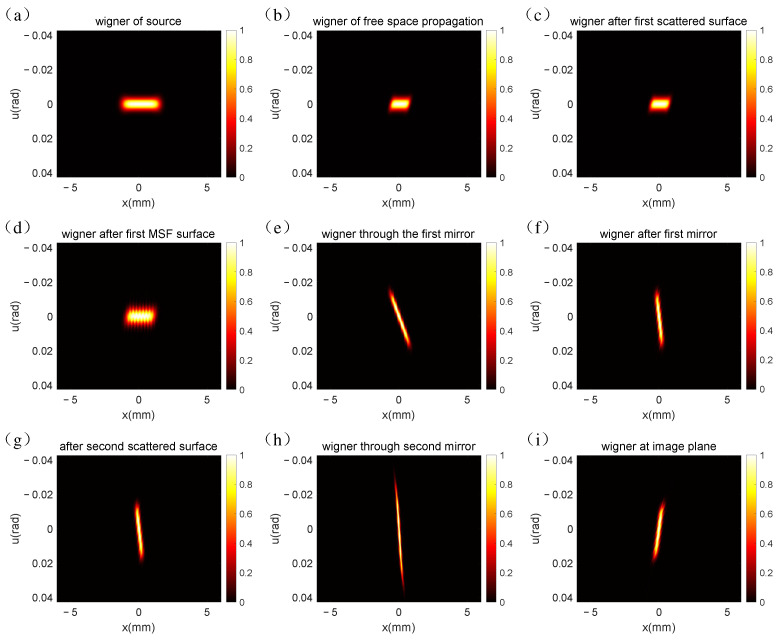
CZT-based Wigner distribution in the RC optical system: (**a**) Wigner distribution of the multi-Gauss Schell model beam; (**b**) Wigner distribution after free space propagation; (**c**) Wigner distribution HSF errors over the surface of the primary mirror; (**d**) Wigner distribution MSF errors over the surface of the primary mirror; (**e**) Wigner distribution of phase modulated by primary mirror; (**f**) free space transmission from primary mirror to secondary mirror; (**g**) Wigner distribution HSF errors over the surface of the secondary mirror; (**h**) Wigner distribution of phase modulated by primary mirror; (**i**) free space transmission from secondary mirror to the image plane.

Similarly, the CZT is used during the propagation process, replacing Equation (8) with Equation (27), and the resulting outcome is shown in Figure 4. To validate the flexibility of the CZT algorithm, the output matrix dimensions were modified. For computational convenience, parameter M in Equation (23) was configured as 512, an integer power of two, and the parameters A and W in Equations (24) and (25) were set as follows:

(30) $$A=e^{j\pi }=-1,\;W=e^{j\pi M}$$

As evidenced in Figure 4, the Wigner distribution based on the CZT aligns with the results obtained from the DFT algorithm. Nevertheless, the Wigner distribution depicted in Figure 4 is more compact compared to that in Figure 3. This discrepancy arises from the fact that we have confined our simulation to the region of interest, thereby modulating the dimensions of the image plane throughout the transmission by fine-tuning the parameters of the CZT. Moreover, owing to the adjustment of the sampling count, the matrix dimensions in Figure 4b–i are 1024 × 512, whereas each matrix in Figure 3 exhibits dimensions of 1024 × 1024. This implies that the utilization of CZT significantly mitigates the demand for computer memory.

**Figure 5:**
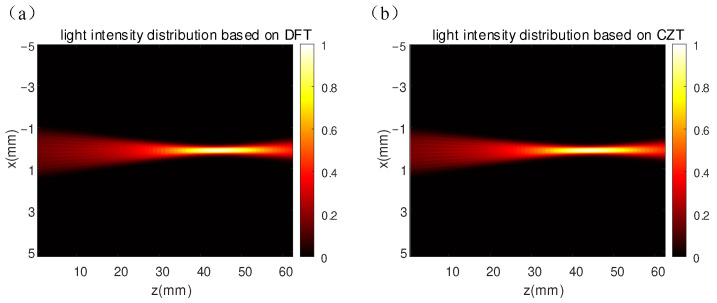
Light intensity distribution of RC telescope system: (**a**) DFT-based light intensity distribution; (**b**) CZT-based light intensity distribution.

Figure 5 was obtained by integrating Equation (3) (light intensity from the Wigner function) after applying the inverse Fourier transform (DFT) to Equation (8) and the inverse CZT (ICZT) to Equation (27), respectively. It can be seen that as the beam travels, the light gradually focuses. However, the beam is not focused at a certain point, but at a distance along the z-axis, because of spherical aberrations in the optical system. Figure 5b is the same as Figure 5a, but it is narrower than Figure 5a because of the selectable nature of the simulation region of CZT.

## 4. Discussion

In our simulation, we meticulously compared the performance of the chirp z-transform (CZT)-based Wigner function propagation method with the conventional discrete Fourier transform (DFT)-based approach. The results revealed that the CZT method not only maintains a high degree of consistency with the DFT method in terms of simulation accuracy, but also demonstrates significant advantages in terms of computational efficiency and memory consumption.

Specifically, in the simulation of the Ritchey–Chrétien telescope system, we quantitatively evaluated the memory usage and runtime of both methods. For the DFT-based simulation, the memory requirement was substantial due to the constraint that the dimensions of the input and output matrices must remain congruent. This led to a large number of redundant calculations and excessive memory allocation. In contrast, the CZT method allowed us to flexibly adjust the number of sampling points and the sampling interval, thereby enabling the targeted simulation of regions of interest within the frequency domain of the optical system. As a result, the memory usage of the CZT-based simulation was reduced by approximately 50% compared to the DFT-based simulation, as evidenced by the matrix dimensions in Figure 4b–i being 1024 × 512, while each matrix in Figure 3 exhibited dimensions of 1024 × 1024.

Regarding runtime, the CZT method also showed a notable improvement. The ability to focus on specific regions of interest and adjust sampling parameters meant that the CZT algorithm could bypass unnecessary computations, leading to a more efficient simulation process. All simulation experiments were conducted on a computer equipped with an Intel(R) Core(TM) i7-10700 CPU with 2.90 GHz and 16 GB of memory. Under the aforementioned hardware configuration, the runtime of the scattering transmission simulation based on DFT is 129.888416 s, while the runtime of simulation based on CZT is 74.454437 s. This means that the run time of CZT-based simulations is 57% of that of DFT-based simulations, without compromising the accuracy of the results.

**Figure 6:**
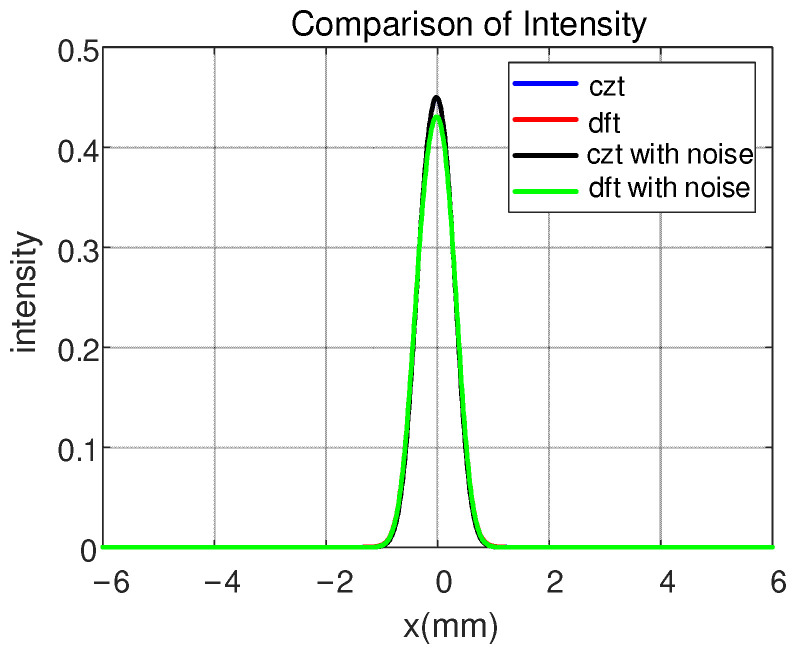
Comparison of light intensity between DFT and CZT methods on the detection plane.

To further describe the differences between the two methods, we plotted the distribution of light intensity on the detection plane, as shown in Figure 6. The two intensity distribution curves in Figure 6 are essentially superimposed, indicating that the results obtained using the CZT method are highly accurate. We also calculated the mean squared error (MSE) between the results obtained from the CZT and DFT methods to quantify the accuracy, and the result is 1.3342 × 10^−5^. The MSE values are low, indicating a high degree of similarity between the two sets of results. This quantitative analysis, combined with the significant improvements in memory usage and runtime, clearly demonstrates the superiority of the CZT method over the existing DFT method in simulating the propagation of partially coherent light within optical systems.

Furthermore, we introduced Gaussian noise with a signal-to-noise ratio (SNR) of 10 dB to the light source. The MSE of the noisy CZT results was found to be 1.4262 × 10^−5^, while the MSE of the noisy DFT results was 4.1188 × 10^−8^. These results indicate that both the CZT and DFT methods are not highly sensitive to noise, as the MSE values of the noisy cases are relatively similar to those of the clean (non-noisy) cases. This demonstrates the robustness of both methods under noisy conditions.

In conclusion, the CZT-based Wigner function propagation method offers enhanced flexibility in simulation, as it allows for variable sampling points and intervals, thereby overcoming the limitations of traditional DFT-based methods. This flexibility not only reduces memory usage but also significantly improves runtime efficiency, making it a more practical and effective approach for studying the transmission characteristics of optical systems. However, some limitations still exist. The current framework assumes paraxial propagation and rotational symmetry, which may not be applicable to non-rotational systems, such as freeform optics. Additionally, the scattering models used in this study are simplified, and real-world surface roughness might require higher-order statistical models for more accurate predictions. Despite these limitations, we believe that the proposed method represents a significant advancement in simulating scattered light propagation and will contribute to more efficient and accurate simulations in future research. These improvements are expected to be of great interest to researchers working on optical systems involving partially coherent light.

## 5. Conclusions

In this paper, we undertake the simulation of the Wigner distribution associated with partially coherent light as it traverses the RC telescope system. The complexity of the optical path transmission is exacerbated by the presence of surface errors on each component within the telescope. Upon comparing the simulation outcomes derived from the CZT and the DFT algorithms, it becomes evident that the Wigner transmission facilitated by the CZT exhibits a heightened degree of flexibility. Even when alterations are made to the number of sampling points and the dimensions of the image plane during the transmission process, accurate simulation results remain attainable. Concurrently, the implementation of CZT leads to a reduction in computer memory requirements during the simulation phase. However, it is important to note that the scope of this paper’s simulation is confined to the paraxial transmission within rotationally symmetric optical systems. The RC telescope’s ability to minimize off-axis aberrations makes it an excellent model for simulating such systems. The findings presented herein can serve as a source of inspiration for future research endeavors focusing on the transmission characteristics of non-rotationally symmetric optical systems.

## Figures and Tables

**Table 1:** The simulation parameters.

Entrance Pupil	Wavelength	Size of Source	First Mirror Coherence Length	Second Mirror Coherence Length
18.75 mm	0.63 μm	5 mm	100 μm	200 μm

## Data Availability

No new data were created or analyzed in this study. Data sharing is not applicable to this article.
